# Effects of Selenium Content on Growth, Antioxidant Activity, and Key Selenium-Enriched Gene Expression in Alfalfa Sprouts

**DOI:** 10.3390/foods13142261

**Published:** 2024-07-18

**Authors:** Yaru Ren, Qian Zhang, Xiang Li, Tianyi Zhang, Daicai Tian, Liang Liu, Xuyan Dong, Zeng-Yu Wang, Maofeng Chai

**Affiliations:** 1 Key Laboratory of National Forestry and Grassland Administration on Grassland Resources and Ecology in the Yellow River Delta, College of Grassland Science, Qingdao Agricultural University, Qingdao 266109, China; 2 Qingdao Key Laboratory of Specialty Plant Germplasm Innovation and Utilization in Saline Soils of Coastal Beach, College of Grassland Science, Qingdao Agricultural University, Qingdao 266109, China; 3 College of Food Science and Engineering, Qingdao Agricultural University, Qingdao 266109, China

**Keywords:** alfalfa sprouts, selenium, differential gene analysis

## Abstract

To enhance the selenium (Se) intake of the general public, the present study implemented biofortification techniques in alfalfa sprouts. Alfalfa sprouts possess unique nutritional value and provide an optimal Se-enriched supplemental Se source. The impact of sodium selenite (Na_2_SeO_3_) on alfalfa shoot germination, shoot length, and biomass was assessed experimentally, and changes in the antioxidant capacity of sprouts treated with optimal Se concentrations were investigated. In addition, the transcriptome of alfalfa sprouts treated with the optimal Na_2_SeO_3_ concentration was sequenced. Gene co-expression networks, constructed through differential gene analysis and weighted gene co-expression network analysis, were used to identify the core genes responsible for Se enrichment in alfalfa sprouts. The findings of the present study offer novel insights into the effects of Se treatment on the nutrient composition of alfalfa sprouts, in addition to introducing novel methods and references that could facilitate production of Se-enriched alfalfa sprouts and associated products.

## 1. Introduction

Selenium (Se) is an essential trace element found in humans and animals. In nature, Se occurs in two main forms: inorganic Se (sodium selenite [Na_2_SeO_3_] and sodium selenate) and organic Se (SeMet). Se exerts a remarkable influence on human health by participating in various metabolic pathways. Its contributions are manifested through organic Se compounds [1,2], which have been identified to possess anti-carcinogenic properties, antioxidant activities [3], and a protective role against immune diseases [4]. In recent years, computational and comparative studies of the various selenium metabolic pathways, selenoproteins, and selenoproteomes (the complete set of selenoproteins) have become more extensive and in depth, e.g., more than 80 selenoprotein families or subfamilies have been reported, with the majority of them being thiol-based oxidoreductases [5]. Selenoproteins are essential for the antioxidant function of Se. They are involved in antioxidant and redox reactions by detoxifying peroxides, regenerating reduced thioredoxin, and reducing oxidized methionine residues [6]. The most mentioned selenoproteins are the glutathione peroxidase group (GPx).

Maintaining recommended Se levels is crucial, as long-term deficiency can pose health risks. Increasing Se content in edible organisms, such as through biofortification, could facilitate Se enrichment and supplementation in the human body [7,8,9]. Se is involved in plant metabolism primarily as inorganic selenate or selenite, which is then reduced to selenide. Selenide is incorporated into selenocysteine (SeCys), an essential step in the biosynthesis of organic selenium compounds. Vegetable plants have the ability to convert inorganic selenium to SeCys and other bioavailable forms, such as selenomethylselenocysteine (MeSeCys), which are more easily absorbed by humans and thus serve as important dietary sources of Se [10].

Germination is a pivotal stage of molecular transformation during plant growth. Sprouts produced through germination can convert macromolecules in seeds into smaller molecules that are more easily digestible, while also reducing anti-nutritional factors [11,12]. Numerous studies have shown that Se is an antioxidant [13,14], and sprouts have the ability to absorb Se in the form of selenite, selenate, and organic Se through their roots [15,16]. Notably, sprouts are marketable products that satisfy consumer demands for nutritious, fresh, and affordable food options, potentially becoming a significant area of research in the food industry [17].

Among various sprout types, alfalfa sprouts possess unique nutritional value and additional benefits. They have been reported to inhibit inflammatory indicators, improve overall antioxidant capacity, and exhibit anti-inflammatory effects [18]. During germination, alfalfa sprouts display a significant increase in isoflavonoids, including soy glucosides, genistein flavonoids, and stilbene, which participate in the flavonoid biosynthetic pathway [19]. Seed germination biofortification is a rapid and effective approach to produce Se-enriched foods [20]. Significant Se accumulation has been observed in alfalfa sprouts when germinated in a selenate solution [21,22]. Therefore, with their distinct flavor, alfalfa sprouts hold promise as a source of Se-enriched supplemental Se foods [23].

The objective of this study was to systematically investigate the effects of Se on the growth and antioxidant properties of alfalfa sprouts. First, our study aimed to determine the optimal concentration of sodium selenite (Na_2_SeO_3_) to promote germination and growth without affecting biomass production. Then, transcriptome sequencing and weighted gene co-expression network analysis (WGCNA) were used to identify key genes associated with Se accumulation. By identifying these genes, this study aimed to elucidate the molecular mechanisms underlying the beneficial effects of Se on alfalfa sprouts, which could shed light on the development of Se-enriched functional foods.

## 2. Materials and Methods

### 2.1. Plant Material and Experimental Design

Alfalfa seeds (“Zhongmu No. 1” cultivar) [24] were used for sprout cultivation. Na_2_SeO_3_ solution was prepared at concentrations of 0, 10, 20, 30, 40, 50, 60, 70, 80, 90, and 100 mg/L [25,26]. The seeds were spread in a flat dish and soaked in the prepared Na_2_SeO_3_ solution for 20 h. Afterward, the seeds were washed three times with distilled water and placed in boxes made of filter paper. Each box was placed in a black plastic tray with grooves at the bottom, and water was added to the tray to maintain the water level at the top of the seeds. The sprouts were cultivated at 20 ± 1 °C under dark condition. Sprouts were harvested after 7 d of growth. After thoroughly washing with distilled water, three replicates of each treatment from three separate test plots were sampled to determine the growth and physiological index.

### 2.2. Determination of Total and Organic Selenium in Sprouts

Samples for Se determination were collected from 11 different concentration treatments, as described in Section 2.1. Three replicates were collected for each concentration treatment. Approximately 0.5 g samples were weighed. Subsequently, 10 mL of nitric acid (70%) and 2 mL of hydrogen peroxide (30% H_2_O_2_) were added, and then the solution was shaken to mix well and digested in a microwave digester. After digestion, the sample was heated to dryness, and then 5 mL of hydrochloric acid (6 mol/L) was added. Thereafter, the solution was heated to transparency, and then 2.5 mL of potassium ferricyanide (100 g/L) was added. The solution was fixed to 10 mL, and the total and inorganic Se of the solution were determined using an atomic spectrometer equipped with a Se hollow cathode lamp. Organic Se concentration was obtained by subtracting inorganic Se concentration from total Se concentration [27].

### 2.3. Determination of Antioxidant Activity

#### 2.3.1. Determination of Scavenging Ability of Alfalfa Sprout Extracts to Inhibit the Transfer of Hydrogen Atoms Using the 2,2-Diphenylpicrylhydrazine (DPPH) Assay

2,2-diphenyl-1-trinitrohydrazine (DPPH) is a stable nitrogen radical with a purple color and an unpaired electron in its structure. When antioxidants are present, they can provide hydrogen atoms or electrons to the DPPH radical, reducing it to a colorless, non-radical form. The DPPH method evaluates antioxidant activity by measuring the change in absorbance before and after sample addition. The DPPH method primarily evaluates the free radical scavenging ability of an antioxidant, specifically the scavenging effect by stabilizing free radicals. This may reflect the direct antioxidant action of the antioxidant, such as neutralizing free radicals by donating hydrogen atoms or electrons.

The DPPH method is used worldwide to quantify free radical scavenging activity. Here, the thirty-minute light avoidance assay mentioned by Rodrigo Scherer [28] was chosen. Three sample replicates were applied for control group (0 mg/L Na_2_SeO_3_) and treatment group (60 mg/L Na_2_SeO_3_), respectively. The absorbance was measured at 517 nm using a spectrophotometer.

A standard curve was first constructed based on the concentration of standard Trolox (x) and its clearance (y).

The standard curve was: y = kx + b = 2.7027x + 3.1474

Calculate the DPPH radical scavenging rate using Equation (1).

(1) $$\begin{array}{c} \mathrm{DPPH}\mathrm{radical}\mathrm{scavenging}\mathrm{rate}\;(\%)\\ \;=[(1-(A\;\mathrm{test}\mathrm{substance}-A\;\mathrm{control}\mathrm{substance})÷A\;\mathrm{blank}\mathrm{substance})\times 100]\%. \end{array}$$

where: A control is the absorbance of the Trolox standard and A blank is the reading of the blank well.

Calculate DPPH radical scavenging capacity from DPPH radical scavenging rate obtained above, according to Equation (2).

(2) $$\begin{array}{c} \mathrm{DPPH}\mathrm{radical}\mathrm{scavenging}\mathrm{capacity}\;(\mu g\;\mathrm{trolox}/g\;\mathrm{weight})\\ \;=[(\mathrm{scavenging}\mathrm{rate}-3.1474)÷2.7027\times V1]÷(V1÷V\times W)\times D \end{array}$$

#### 2.3.2. Determination of Scavenging Ability of Alfalfa Sprout Extracts on Hydroxyl Radicals Using the Salicylic Acid Method

Hydroxyl radicals are highly reactive free radicals that are normally produced in the body by the reaction of metal ions (e.g., iron ions) and H_2_O_2_. The salicylic acid method is used to measure the scavenging ability of hydroxyl radicals. In this method, hydroxyl radicals react with salicylic acid to form 2,3-dihydroxybenzoic acid, which can be quantified through absorbance measurements. If antioxidants are present, they will scavenge the hydroxyl radicals and reduce the formation of 2,3-dihydroxybenzoic acid, thereby reducing the absorbance. Since hydroxyl radicals are highly active and harmful free radicals in cells, this method reflects the role of antioxidants in protecting against intracellular oxidative damage.

Hydroxyl radical scavenging activity was determined according to Dagmara’s method [29] with minor modification. Three sample replicates were applied for control group (0 mg/L Na_2_SeO_3_) and treatment group (60 mg/L Na_2_SeO_3_), respectively. Each sample replicate weighed 0.1 g. Firstly, 300 μL FeSO_4_ (9 mmol/L), 300 μL H_2_O_2_, and 300 μL sample extract were mixed. After shaking, the mixture was added to 300 μL of ethanol salicylate (9 mmol/L) and incubated at 37 °C for 30 min. The absorbance was measured at 510 nm. The scavenging rate was calculated according to Equation (3).

Hydroxyl radical scavenging rate (%) = [A blank − (A test − A control)] ÷ A blank × 100%.
(3)

where: A control is the absorbance of the Trolox standard and A blank is the reading of the blank well.

#### 2.3.3. Determination of Antioxidant Activity by Selenium-Related Enzymes

The activities of glutathione peroxidase (GPx) and catalase (CAT) as well as the malondialdehyde (MDA) level were measured.

GPx activity was determined using the NADPH method, which measures enzyme activity by monitoring the change in absorbance upon oxidation of NADPH to NADP+. In this process, GPx catalyzes the reduction of glutathione (GSH) by peroxides, resulting in the formation of oxidized glutathione (GSSG). The reduction of GSSG back to GSH by glutathione reductase (GR) consumes NADPH, causing a reduction in absorbance that indicates GPx activity.

CAT is widely found in animals, plants, microorganisms and cultured cells and is the predominant H_2_O_2_-scavenging enzyme with an important role in the reactive oxygen species scavenging system. Hydrogen peroxide can oxidize MoO_4_^2−^ to MoO_5_^2−^, whereby MoO_5_^2−^ accepts the electron from the hydroxide to form a bond, and immediately dehydrates and condenses between molecules, resulting in a stable yellow complex (H_2_MoO_4_·XH_2_O)n with a strong absorption peak at 405 nm, and the absorbance value and concentration of hydrogen peroxide are linearly related. The absorbance at 405 nm of the remaining hydrogen peroxide in the system can be determined to reflect the catalytic activity of CAT.

In addition, malondialdehyde (MDA), although not an antioxidant per se, is a marker of lipid peroxidation and oxidative stress. Its measurement is often used to assess the extent of oxidative damage and the protective effects of selenium and other antioxidants. Oxygen free radicals act on the unsaturated fatty acids of lipids to produce lipid peroxides, which are gradually broken down into a complex series of compounds, including MDA, the level of which can be measured to determine the degree of lipid oxidation.

The activities of the above enzymes were performed according to the methods described in the purchased kits (see Table S1 for kit descriptions). The 60% selenium treatment concentration was taken as the treatment group and no treatment as the CK group; three sample replicates were taken from each group and each sample weighed 0.1 g.

### 2.4. Transcriptomic Analysis

Total RNA was extracted from the samples after freezing in liquid nitrogen. mRNA was enriched, fragmented, and reverse transcribed to cDNA. cDNA library construction and subsequent sequencing were performed at Wuhan MetWare Biotechnology Co., Ltd. (Wuhan, China), and the quality of the cDNA libraries was assessed using Qubit 2.0 fluorometer, Agilent 2100, and qRT-PCR. Sequencing was performed on the Illumina HiSeq platform. Clean reads were obtained after filtering the raw data, checking the sequencing error rate, and checking the GC content distribution. The clean reads were then mapped to the reference genome using HISAT2 to obtain information on the location of the reference genome as well as information on specific sequence features of the sequenced samples. Reads were counted for genes in each sample using the mapped reads, and then the gene count results for all samples were assembled. DESeq2 was used to analyze differentially expressed genes (DEGs). Kyoto Encyclopedia of Genes and Genomes (KEGG2), Gene Ontology (GO3), and Clusters of Orthologous Groups of Proteins (COG4) were used to analyze DEGs. COG4 was used for functional annotation and enrichment analysis of DEGs (see Table S2 for descriptions of gene lists).

### 2.5. Construction of Gene Co-Expression Network Using WGCNA

The WGCNA algorithm is a typical systems biology algorithm for constructing gene co-expression networks. It is based on high-throughput gene messenger RNA (mRNA) expression data and is commonly used world-wide in the biomedical field. The WGCNA algorithm assumes that the gene network has a scale-free distribution; defines the gene co-expression correlation matrix, the neighborhood function formed by the gene network; calculates the dissimilarity coefficients of the different nodes; and constructs a hierarchical clustering tree, accordingly. Different branches of the clustering tree represent different gene modules, with a high degree of gene co-expression within modules and a low degree of gene co-expression across modules. Finally, the association between the modules and specific phenotypes was explored to identify target genes and gene networks.

### 2.6. Real-Time Quantitative Fluorescence PCR (qRT-PCR) Analysis

The reliability of the RNA-seq results was verified using qRT-PCR. Four genes were selected for qRT-PCR based on transcriptome results, and primers were designed using the Primer3 program (Table 1). Reverse transcription was performed using the same RNA samples as those used for RNA-seq, and qRT-PCR was performed using ChamQ SYBR Color qPCR Master Mix (Nanjing Vazyme Biotech Co., Ltd., Nanjing, China). Relative expression levels were calculated using the 2^-ΔCT method. Three biological replicates were used, and three technical replicates were performed for each.

## 3. Results and Discussion

### 3.1. Growth and Physiological Characteristics of Alfalfa Sprouts

**Figure 1:**
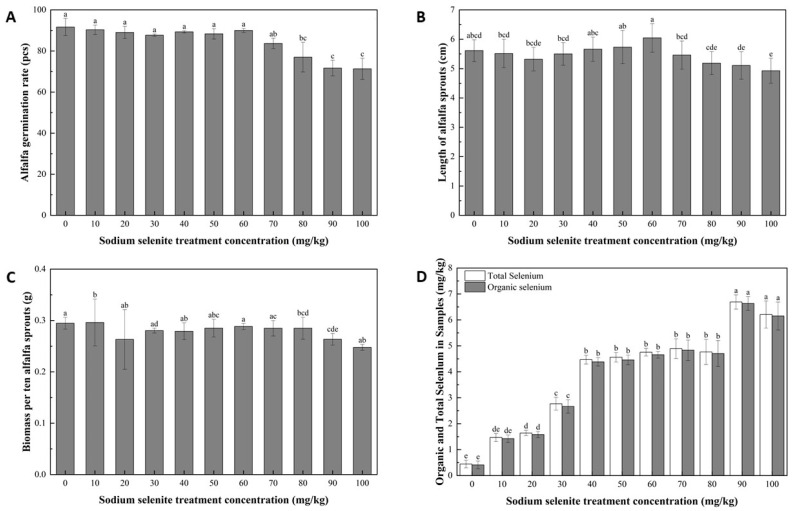
Growth and physiological characteristics of alfalfa sprouts. (**A**) Effect of selenium concentration on germination percentage of alfalfa seeds after a 7-day germination cycle. Analysis of variance (ANOVA) was performed using Origin, and different letters indicate statistically significant differences. The germination data of each concentration treatment were collected from 100 alfalfa seeds of each replicate with three replicates. (**B**) Effect of selenium concentration on alfalfa shoot length (excluding root and cotyledon parts) after a 7-day germination cycle. Analysis of variance (ANOVA) was performed using Origin; different letters indicate statistically significant differences; the data were presented as mean. (**C**) Effect of selenium concentration on the biomass of alfalfa sprouts after a 7-day germination cycle. Analysis of variance (ANOVA) was performed using Origin; different letters indicate statistically significant differences. (**D**) Total and organic selenium content of samples treated with varying selenium concentrations; different letters indicate statistically significant differences.

Following a 7-day seed germination cycle under different concentrations of Na_2_SeO_3_, seed germination rate, shoot length, and sprout biomass were analyzed. As shown in Figure 1A, low doses of Se did not inhibit germination, and the germination rate exhibited negligible effects of Se treatments until the concentration exceeded 60 mg/L, after which it declined gradually and significantly. The length of sprouts was determined by measuring six sprouts from each group, with three replicates for each concentration treatments. The results exhibited an increasing trend in sprout length for concentrations ranging from 0 to 60 mg/L, followed by a decreasing trend from 60 to 100 mg/L (Figure 1B). Biomass measurements were taken from groups of 10 plants per replicate, with three replicates for each concentration. Similarly, a more significant reduction in biomass was observed once the concentration of Na_2_SeO_3_ exceeded 60 mg/L (Figure 1C). As shown in Figure 1D, exogenous Se concentration had a significant impact on the total organic Se content of the alfalfa sprouts. With an increase in treatment concentration, the Se content in the sprouts also increased. Specifically, after the 60 mg/L Na_2_SeO_3_ soaking treatment, the Se content in the sprouts was 8.64-fold higher than that in the control, indicating successful enrichment of Se in the sprouts. Overall, our comprehensive analysis indicated that the most suitable treatment concentration for optimal growth and the highest Se content in the alfalfa sprouts was 60 mg/L of Na_2_SeO_3_. Thus, the optimal Na_2_SeO_3_ concentration for subsequent experiments was determined to be 60 mg/L.

**Figure 2:**
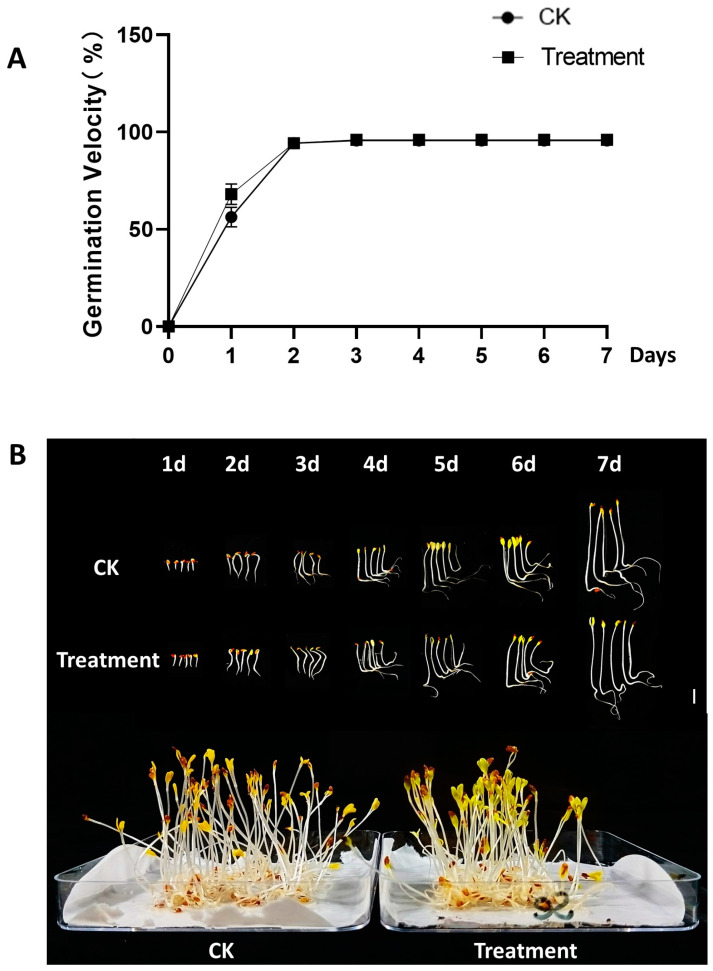
The seed germination velocity (**A**) and the appearance of representative alfalfa sprouts (**B**) grown from 1 to 7 days at Se concentrations of 0 (CK) and 60 mg/L (Treatment). Scale bar = 1 cm. Alfalfa sprouts in the transparent dish were grown for 7 days in the black tray and photographed. The alfalfa seeds were measured for growth velocity in three replicates with 50 seeds per replicate.

To more comprehensively evaluate the effect of 60 mg/L Na_2_SeO_3_ soaking treatment on germination of alfalfa seeds, we analyzed the velocity of germination and imaged the growth of alfalfa sprouts. On the first day of germination, Se-treated seeds showed a statistically significant increase in their germination rate compared with the untreated control group as shown in Figure 2. On the second day, the germination rate was more than 80% in both groups and there was no significant difference throughout the rest of the 7-day germination cycle. The rest of the physiological indicators were not significantly affected during the 0–7 days of growth. These results indicated that the treatment of the chosen Se concentration had a positive effect on the early growth of sprouts, especially improving the germination potential. It was also confirmed that 60 mg/L Na_2_SeO_3_ treatment had no adverse effect on the growth of the sprouts.

### 3.2. Analysis of Antioxidant Capacity

#### 3.2.1. Hydroxyl Radical Scavenging Capacity

**Figure 3:**
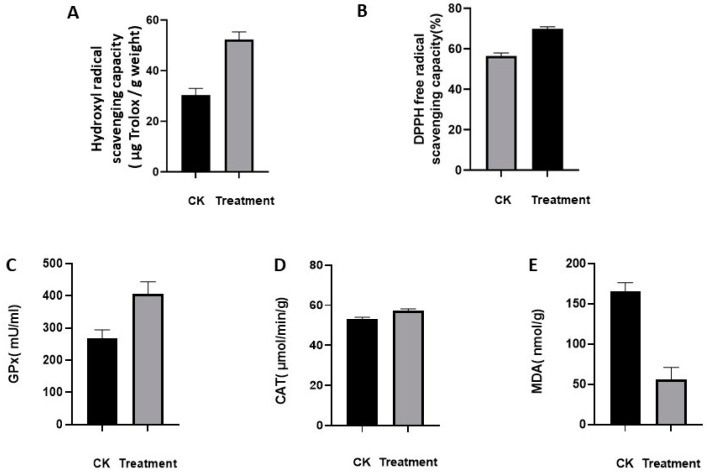
Analysis of the antioxidant capacity of selenium-enriched alfalfa sprouts. (**A**) Hydroxyl radical scavenging rate. (**B**) DPPH radical scavenging rate. (**C**) Changes in GPx content. (**D**) Changes in CAT content. (**E**) Changes in MDA content in CK (0 mg/L Na_2_SeO_3_) and Treatment (60 mg/L Na_2_SeO_3_) groups.

The hydroxyl radical scavenging capacity was determined in sprouts treated with Na_2_SeO_3_ concentrations of 0 mg/L (CK) and 60 mg/L (Treatment), as described in Section 2.3. As shown in Figure 3A, the average hydroxyl radical scavenging rate was 30% for alfalfa sprouts not treated with Se and 52% for sprouts treated with the optimal Se concentration. The results indicated that the hydroxyl radical scavenging capacity of Se-enriched alfalfa sprouts was significantly higher than that of the control group. Thus, the antioxidative capacity of Se-enriched alfalfa sprouts were increased.

#### 3.2.2. DPPH-Free Radical Scavenging Capacity

The DPPH radical scavenging capacity was determined at Na_2_SeO_3_ concentrations of 0 mg/L (CK) and 60 mg/L (Treatment), as described in Section 2.3. The results showed that the average DPPH radical scavenging rate was 56% for alfalfa sprouts not treated with Se and 69% for sprouts treated with the optimal Se concentration (Figure 3B). This indicates that the DPPH radical scavenging ability of Se-enriched alfalfa sprouts was significantly higher than that of the control group. Therefore, the antioxidant properties of Se-enriched alfalfa sprouts were enhanced.

#### 3.2.3. Antioxidant Capacity of Selenium-Related Enzymes

The results, as shown in Figure 3C–E, indicated that the Se-treated group exhibited a significant increase in GPx activity. This suggested that selenoproteins possessed superior antioxidant capacity upon Se treatment. Moreover, CAT activity was slightly increased compared to those in the CK group, indicating that Se treatment effectively scavenged reactive oxygen species. Furthermore, MDA, a biomarker that measures oxidative stress, was significantly reduced, demonstrating that Se treatment effectively mitigated lipid peroxidation through antioxidant mechanism, thereby reducing the MDA production. The comprehensive assessment of these interconnected indicators led to the conclusion that the Se-treated samples exhibited superior antioxidant properties.

### 3.3. Analysis of Differentially Expressed Genes (DEGs)

**Figure 4:**
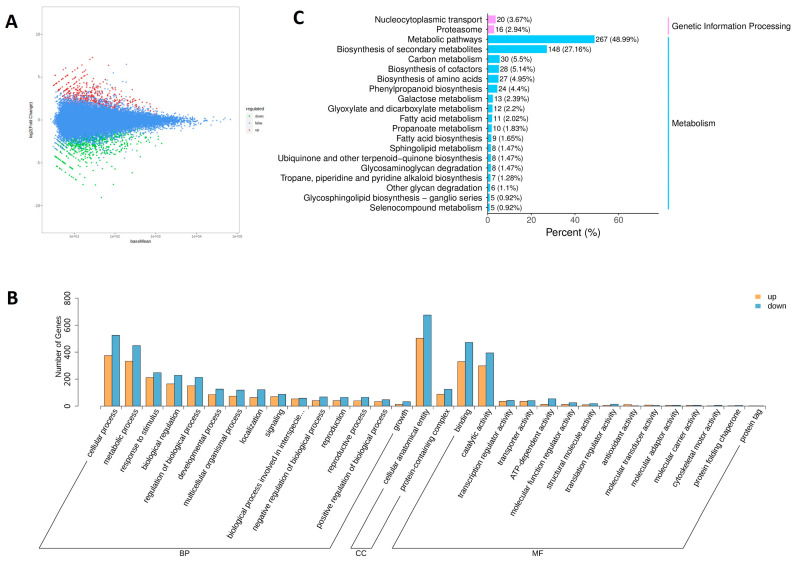
Transcriptomic overview of selenium-induced gene expression in selenium-enriched alfalfa sprouts. (**A**) Differential gene MA map. (**B**) GO categorization statistics following up- and down-regulation of differentially expressed genes. (**C**) KEGG pathway of DEGs.

To investigate the mechanisms underlying the changes in Se-enriched alfalfa sprouts, a transcriptome sequencing assay was performed. A total of 275,512,432 original sequences were obtained, which were then filtered and processed, resulting in 268,605,598 clean sequences. The mean Q20 values for the CK group and the treatment group were 98.06% and 98.09%, respectively, whereas the mean Q30 values were 94.18% and 94.24%, respectively. The relative GC content was 42.42% for the CK group and 42.54% for the Treatment group. These results indicate that the transcriptome sequencing data had good quality and high accuracy. A total of 1586 DEGs were identified, with 904 downregulated and 682 upregulated, accounting for 57% and 43% of the total DEGs, respectively (Figure 4A).

### 3.4. Gene Ontology (GO) Enrichment Analysis of DEGs

The DEGs were annotated using GO functions and classified into 19 functional subclasses. GOs were divided into three categories: molecular function (MF), biological process (BP), and cellular component (CC). The top 15 GO terms (or all if there were <15) were selected in each category based on the number of genes in the annotations. The classification histogram showed the distribution of upregulated and downregulated DEGs for each functional subclass (Figure 4B). BPs were classified into 15 functional subclasses, mainly involving cellular and metabolic processes. CC had two functional subclasses, namely cellular structural bodies and protein-containing complexes. MF had 15 functional subclasses, with catalytic activity and binding being the main annotations.

### 3.5. KEGG Enrichment Analysis of DEGs

To explore the metabolic pathways involved in the DEGs of alfalfa sprouts, the DEGs were compared and analyzed using the KEGG database. The KEGG enrichment results were divided into two categories: metabolism and genetic information processing. The metabolism category exhibited more enrichment results than the genetic information processing category (Figure 4C). The KEGG analysis revealed a significant involvement of the metabolic pathway, followed by secondary metabolite biosynthesis. Specific pathways such as phenylpropanoid biosynthesis, amino acid biosynthesis, amino sugar and nucleotide sugar metabolism, and flavonoid biosynthesis were also identified. In the genetic information processing category, nucleocytoplasmic transport and proteasome pathways were found to be involved.

### 3.6. DEGs Involved in Selenium Metabolism

**Figure 5:**
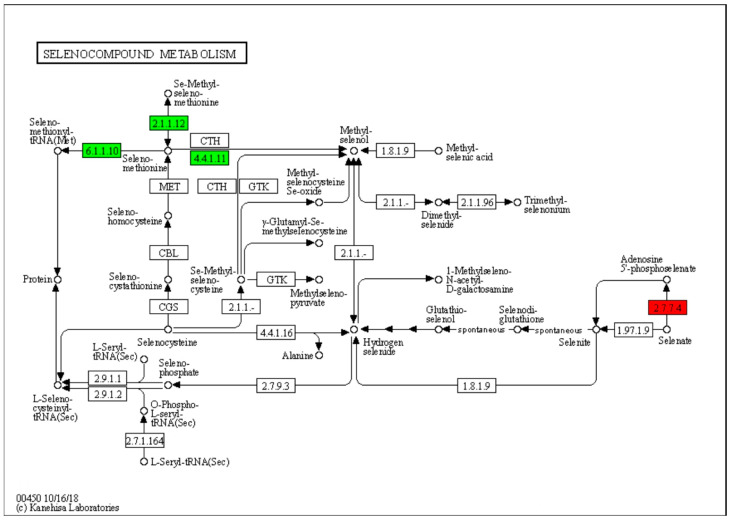
DEGs in selenium metabolic pathways of KEGG metabolic pathway map. Green indicates down-regulated genes, red indicates up-regulated genes, and white indicates no significant change in gene expression levels.

The KEGG pathway analysis of the top 20 enrichment factors showed that metabolism-related DEGs were the most significantly enriched among the treatments. Se biofortification in plants occurs through the sulfur (S) metabolism pathway due to the chemical similarity between Se and S [15,30]. The S metabolism pathway is responsible for converting S and Se into various organic compounds, including selenoproteins and other organic selenides. The KEGG map reveals the complex metabolic network of Se in living organisms and its critical role in maintaining the health and function of living organisms. The Se compounds of metabolic pathways in organisms are shown in the KEGG map. Based on the KEGG analysis, four DEGs were identified in the S and Se pathways: *3′-phosphoadenosine 5′-phosphosulfate synthase* (*PAPSS*) displayed upregulated expression, while *methionine S-methyltransferase* (*SMM*), *methionyl-tRNA synthetase* (*MARS*), and *methionine gamma-lyase* (*MGL*) showed downregulated expression (Figure 5).

### 3.7. qRT-PCR Validation

**Figure 6:**
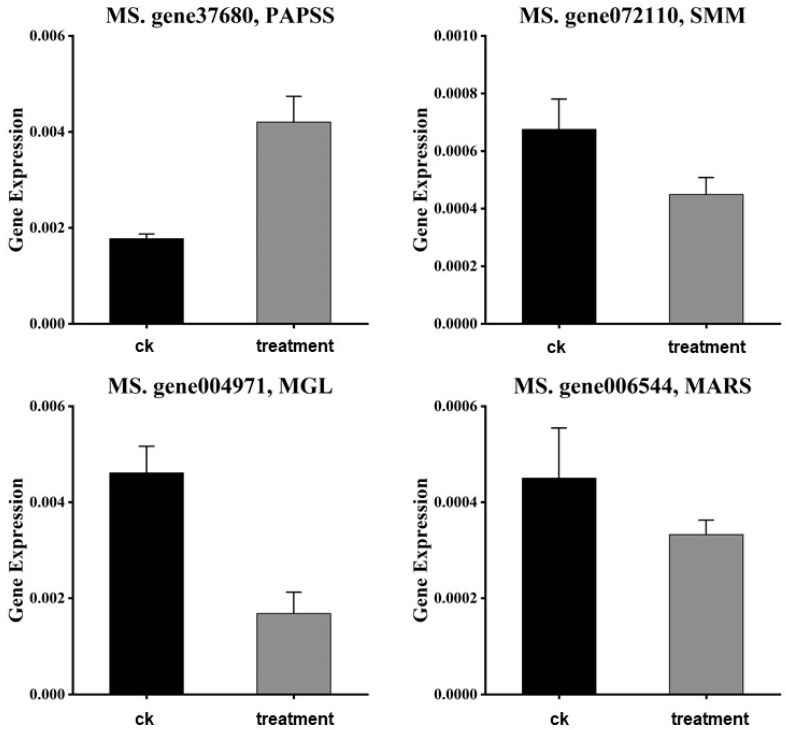
The qRT-PCR analysis of the genes related to selenium metabolic pathways in selenium-enriched alfalfa sprouts.

The expression patterns of the four selected genes (PAPSS, SMM, methionyl-tRNA synthetase, and MGL) were validated using qRT-PCR (Figure 6). The qRT-PCR results confirmed the results of the RNA-Seq data, supporting the involvement of the genes in Se metabolism in plants.

### 3.8. Gene Correlation Network Construction

WGCNA was performed to identify gene modules based on their expression correlations. Each color in the corresponding figure indicates the corresponding gene in the clustering tree belonging to the same module. If some genes have similar expression trends in a physiological process or in different tissues, the genes may be functionally related and can be defined as a module. WGCNA utilizes weighted values of the correlation coefficients, specifically the Nth power of the gene correlation coefficient. The soft threshold selection diagram and module-level clustering tree diagram can be seen in Figure S1.

The intermodule-correlation heatmap can be divided into two parts: the upper and lower parts. The upper part clusters modules based on their eigenvalues (eigengenes). The vertical coordinates represent the degree of dissimilarity of the nodes, and each row and column in the lower part of the graph represents a module. The darker (redder) the square, the stronger the correlation, and the lighter the square, the weaker the correlation. 

The correlation intensity of gene expression modules with selenium enrichment under treatment was evaluated. The yellow and magenta modules displayed a significant positive correlation with the Treatment group, as indicated by their intensified red coloration, which is attributed to an increased co-expression related to selenium incorporation. This suggests these modules may play a pivotal role in the response to Se enrichment.

### 3.9. Analysis of the Regulation of Key Gene Expression Modules

**Figure 7:**
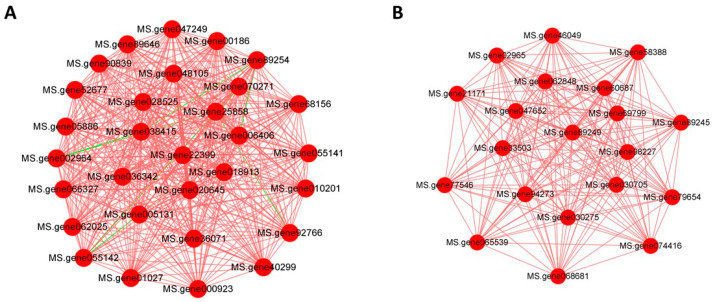
Analysis of the regulation of key gene expression modules. (**A**) Correlation network diagram of key genes in the yellow module. (**B**) Correlation network diagram of key genes in the magenta module.

Key genes related to Se metabolism were identified from the two modules by combining relevant literature. Thirty key genes were screened in the yellow module and twenty key genes were screened in the magenta module, as shown in Table 2. The screened genes were analyzed for correlation. Figure 7A,B show the correlation network diagrams of the key genes. MS.gene22399 (MATs) and MS.gene89249 (GST) were identified as the most highly correlated genes, also known as hub genes, in the module. MS.gene22399 encodes for S-adenosylmethionine synthase (MATs), which is responsible for producing S-adenosylmethionine (SAM) and cofactors required for various methylation reactions [31]. SAM can be converted metabolically to selenocysteine, thereby promoting Se accumulation. A common feature of Se-accumulating in plants is an increased tolerance to Se along with the synthesis of Se-containing compounds such as cysteine, γ-glutamylcysteine or selenocystathionine [32,33]. Therefore, MATs are thought to be a factor involved in both Se accumulation and Se tolerance.

MS.gene89249 encodes a glutathione S-transferase (GST). GSTs in plants are involved in the detoxification of exogenous substances as well as endogenous reaction products of cellular metabolism and play an important role in phytoremediation and plant detoxification [34,35,36]. Previous studies have shown that Se can enhance the antioxidant capacity of plants through multiple systems. It has been observed that GST levels increase with an increase in antioxidant capacity to mitigate Se toxicity. Additionally, Se supplementation notably boosts soluble GST activity, indicating that GST levels rise in the presence of Se and are closely linked to the antioxidant mechanism of Se [37,38].

## 4. Discussion

In nature, plants can utilize their Se accumulation capacity to enhance their ecological advantages. It has been demonstrated that the use of exogenous Se can promote the growth of salt-stressed tomato seedlings [22]. Moreover, a low dosage of Se (3.2 mg/L) has been found to enhance seedling emergence, plant height, biomass, and chlorophyll levels [23]. Therefore, we studied the effects of Se content on the growth, antioxidant activity, and identification of key Se-enriched genes in alfalfa sprouts. The results of the present study showed that Se-enriched alfalfa sprouts had increased antioxidant capacity compared to ordinary sprouts, as indicated by the higher DPPH and hydroxyl radical scavenging capacities. This is consistent with previous studies that have reported the antioxidant properties of Se-enriched plants [1]. The increased antioxidant capacity of Se-enriched sprouts can be attributed to the presence of selenoproteins, which have been shown to have strong antioxidant properties [2]. The upregulation of certain genes involved in Se metabolism, such as PAPSS, SMM, and MGL, also suggests that the increased antioxidant capacity may be related to the synthesis and metabolism of selenoproteins.

Furthermore, the downregulation of genes such as SMM, methionyl-tRNA synthetase, and MGL indicates potential regulatory mechanisms for Se metabolism. SMM has been shown to contribute to S transport in plants and may play a role in Se transport as well [20]. Methionyl-tRNA synthetase is involved in the synthesis of selenocysteine, an essential component of selenoproteins [27]. Methionine gamma-lyase is involved in S metabolism and may also be involved in the S metabolism of sprouts [21]. Further studies are required to elucidate the specific roles of the genes in Se metabolism and accumulation in alfalfa sprouts.

The validation of the selected genes using qRT-PCR provides further support for their involvement in Se metabolism. The consistent expression patterns between RNA-Seq analysis and qRT-PCR analysis indicate the reliability of the transcriptome sequencing data and the potential significance of the genes in Se accumulation.

WGCNA revealed gene modules that are highly correlated with Se-enriched alfalfa sprouts. The modules may contain core genes that play important roles in Se enrichment. The identification of these hub genes provides valuable information for further studies on the mechanisms underlying Se metabolism and accumulation in plants.

The accumulation of Se in plants can vary between different species and growth stages. Alfalfa, for example, has been found to accumulate more Se in its young leaves and stems than in its older leaves and roots [39]. Various enzymes in the Se pathway could play important roles in Se accumulation and transformation. In the selenometabolism pathway, seleno-reductase plays a crucial role in converting inorganic Se, such as selenate, into the organic form of Se, making it more available for plant uptake and utilization [40]. Additionally, in the S metabolism pathway, increased uptake of S via transport proteins, known as SULTRs, along with overexpression of enzymes in the Se metabolism pathway, can increase the production of non-toxic Se compounds in plants [41].

To enhance the ability to metabolize Se, it would be beneficial to further express these enzymes in alfalfa sprouts. This would facilitate Se conversion and utilization in the plants. In other words, by increasing the expression of these enzymes, the bioconversion of Se can be improved, potentially leading to higher Se accumulation and utilization in alfalfa sprouts.

Although this study provides some insights into Se accumulation in alfalfa sprouts, several limitations must be recognized in order to fully interpret the results. Light and moisture were artificially controlled during sprout cultivation, so it is inevitable that there were some effects of different conditions on the physiological status of the sprouts. Although we controlled for the Se concentration as a variable, there may be other confounding factors that were not included in the analysis. A deeper and more comprehensive analysis will be conducted in the future with the expectation that these limitations will be addressed, and a more robust approach will be used to further explore the issue.

## 5. Conclusions

Overall, the present study introduces a novel method of production of Se-enriched alfalfa sprouts and provides valuable insights into the mechanisms underlying Se accumulation and metabolism in alfalfa sprouts. The upregulation of genes involved in the S metabolism pathway and the downregulation of genes related to Se metabolism suggest potential regulatory mechanisms for Se accumulation and utilization in plants. The validation of the selected genes using qRT-PCR provides further support for their involvement in Se metabolism. WGCNA provides a framework for understanding the co-expression patterns of genes and identifying modules that may play important roles in Se-enriched plant growth. Further studies are required to elucidate the functions of these genes and their roles in Se accumulation and to optimize Se biofortification strategies in alfalfa sprouts to enhance their antioxidant capacity and potential health benefits.

## Figures and Tables

**Table 1:** Primers.

Primer Name	Primer Sequence
PAPSS37680 F	TGATCCATCAAGGCCTCAGG
PAPSS37680 R	CAACTAGCACCTTCCAGCCA
SMM072110 F	TCTCACCTCGGCTTGCAATT
SMM072110 R	TCACCACCACTTGAGGCTTC
MGL004971 F	CCTAGAAGACCATCCACATCATGA
MGL004971 R	GTGCTACTTCCAGAACAAGACATAA
MARS006544 F	GTTGAGTTGGTTGAACCGCC
MARS006544 R	AATGCCAGCTGATGTGGTCA

**Table 2:** Module Key Genes and KEGG Functional Annotation.

Module Colors	Gene ID	KEGG
yellow	MS.gene000923	K22366 thioredoxin reductase-like selenoprotein T|(GenBank) selT/selW/selH selenoprotein domain protein (A)
yellow	MS.gene00186	K00382 dihydrolipoamide dehydrogenase [EC:1.8.1.4]|(GenBank) dihydrolipoamide dehydrogenase (A)
yellow	MS.gene002964	K05907 adenylyl-sulfate reductase (glutathione) [EC:1.8.4.9]|(GenBank) 5′-adenylylsulfate reductase (A)
yellow	MS.gene005131	K12859 U5 snRNP protein, DIM1 family|(GenBank) mRNA splicing factor, thioredoxin-like U5 snRNP protein (A)
yellow	MS.gene006406	K08967 1,2-dihydroxy-3-keto-5-methylthiopentene dioxygenase [EC:1.13.11.53 1.13.11.54]|(GenBank) 1,2-dihydroxy-3-keto-5-methylthiopentene dioxygenase (A)
yellow	MS.gene010201	K23870 putative pectin methyltransferase [EC:2.1.1.-]|(GenBank) S-adenosylmethionine-dependent methyltransferase, putative (A)
yellow	MS.gene01027	K05907 adenylyl-sulfate reductase (glutathione) [EC:1.8.4.9]|(GenBank) 5′-adenylylsulfate reductase (A)
yellow	MS.gene018913	K03671 thioredoxin 1|(GenBank) thioredoxin H-type 1 protein (A)
yellow	MS.gene020645	K03671 thioredoxin 1|(GenBank) thioredoxin (A)
yellow	MS.gene028525	K07305 peptide-methionine (R)-S-oxide reductase [EC:1.8.4.12]|(GenBank) methionine sulfoxide reductase B 2 (A)
yellow	MS.gene036342	K00559 sterol 24-C-methyltransferase [EC:2.1.1.41]|(GenBank) S-adenosyl-methionine-sterol-C- methyltransferase (A)
yellow	MS.gene038415	K07304 peptide-methionine (S)-S-oxide reductase [EC:1.8.4.11]|(GenBank) peptide methionine sulfoxide reductase family protein (A)
yellow	MS.gene047249	K08247 methionine S-methyltransferase [EC:2.1.1.12]|(GenBank) methionine S-methyltransferase (A)
yellow	MS.gene048105	K00799 glutathione S-transferase [EC:2.5.1.18]|(GenBank) glutathione S-transferase, amino-terminal domain protein (A)
yellow	MS.gene055141	K00799 glutathione S-transferase [EC:2.5.1.18]|(GenBank) glutathione S-transferase, amino-terminal domain protein (A)
yellow	MS.gene055142	K00799 glutathione S-transferase [EC:2.5.1.18]|(GenBank) glutathione S-transferase, amino-terminal domain protein (A)
yellow	MS.gene05886	K01759 lactoylglutathione lyase [EC:4.4.1.5]|(GenBank) lactoylglutathione lyase-like protein (A)
yellow	MS.gene062025	K00789 S-adenosylmethionine synthetase [EC:2.5.1.6]|(GenBank) S-adenosylmethionine synthase-like protein (A)
yellow	MS.gene066327	K23870 putative pectin methyltransferase [EC:2.1.1.-]|(GenBank) S-adenosylmethionine-dependent methyltransferase, putative (A)
yellow	MS.gene070271	K19306 18S rRNA (guanine1575-N7)-methyltransferase [EC:2.1.1.309]|(GenBank) S-adenosyl-L-methionine-dependent methyltransferase (A)
yellow	MS.gene22399	K00789 S-adenosylmethionine synthetase [EC:2.5.1.6]|(GenBank) S-adenosylmethionine synthase-like protein (A)
yellow	MS.gene25858	K11820 N-hydroxythioamide S-beta-glucosyltransferase [EC:2.4.1.195]|(GenBank) UDP-glucosyltransferase family protein (A)
yellow	MS.gene36071	K03671 thioredoxin 1|(GenBank) thioredoxin M-type protein (A)
yellow	MS.gene40299	K01759 lactoylglutathione lyase [EC:4.4.1.5]|(GenBank) lactoylglutathione lyase-like protein (A)
yellow	MS.gene52677	K03671 thioredoxin 1|(GenBank) thioredoxin (A)
yellow	MS.gene68156	K01244 5′-methylthioadenosine nucleosidase [EC:3.2.2.16]|(GenBank) 5′-methylthioadenosine/S-adenosylhomocysteine nucleosidase (A)
yellow	MS.gene89254	K00799 glutathione S-transferase [EC:2.5.1.18]|(GenBank) glutathione S-transferase, amino-terminal domain protein (A)
yellow	MS.gene89646	K07304 peptide-methionine (S)-S-oxide reductase [EC:1.8.4.11]|(GenBank) peptide methionine sulfoxide reductase family protein (A)
yellow	MS.gene90839	K21888 glutathione dehydrogenase/transferase [EC:1.8.5.1 2.5.1.18]|(GenBank) glutathione S-transferase (A)
yellow	MS.gene92766	K03671 thioredoxin 1|(GenBank) thioredoxin (A)
magenta	MS.gene02965	K00799 glutathione S-transferase [EC:2.5.1.18]|(GenBank) glutathione S-transferase, amino-terminal domain protein (A)
magenta	MS.gene030275	K03671 thioredoxin 1|(GenBank) thioredoxin H-type 1 protein (A)
magenta	MS.gene030705	K15111 solute carrier family 25 (mitochondrial S-adenosylmethionine transporter), member 26|(GenBank) S-adenosylmethionine carrier protein (A)
magenta	MS.gene047652	K01265 methionyl aminopeptidase [EC:3.4.11.18]|(GenBank) methionine aminopeptidase 1B (A)
magenta	MS.gene062848	K00799 glutathione S-transferase [EC:2.5.1.18]|(GenBank) glutathione S-transferase, amino-terminal domain protein (A)
magenta	MS.gene065539	K00658 2-oxoglutarate dehydrogenase E2 component (dihydrolipoamide succinyltransferase) [EC:2.3.1.61]|(GenBank) 2-oxoacid dehydrogenase acyltransferase family protein (A)
magenta	MS.gene068681	K00799 glutathione S-transferase [EC:2.5.1.18]|(GenBank) glutathione S-transferase, amino-terminal domain protein (A)
magenta	MS.gene074416	K00121 S-(hydroxymethyl)glutathione dehydrogenase/alcohol dehydrogenase [EC:1.1.1.284 1.1.1.1]|(GenBank) zinc-binding alcohol dehydrogenase family protein (A)
magenta	MS.gene21171	K22966 sulfate adenylyltransferase (ADP)/adenylylsulfatase [EC:2.7.7.5 3.6.2.1]|(GenBank) scavenger mRNA decapping enzyme carboxy-term-binding protein (A)
magenta	MS.gene33503	K22366 thioredoxin reductase-like selenoprotein T|(GenBank) selT/selW/selH selenoprotein domain protein (A)
magenta	MS.gene46049	K00799 glutathione S-transferase [EC:2.5.1.18]|(GenBank) glutathione S-transferase, amino-terminal domain protein (A)
magenta	MS.gene58388	K01265 methionyl aminopeptidase [EC:3.4.11.18]|(GenBank) methionine aminopeptidase 2B-like protein (A)
magenta	MS.gene60687	K03671 thioredoxin 1|(GenBank) thioredoxin M-type protein (A)
magenta	MS.gene69799	K03671 thioredoxin 1|(GenBank) thioredoxin (A)
magenta	MS.gene77546	K11820 N-hydroxythioamide S-beta-glucosyltransferase [EC:2.4.1.195]|(GenBank) UDP-glucosyltransferase family protein (A)
magenta	MS.gene79654	K03671 thioredoxin 1|(GenBank) thioredoxin H2-like protein (A)
magenta	MS.gene89245	K00799 glutathione S-transferase [EC:2.5.1.18]|(GenBank) glutathione S-transferase (A)
magenta	MS.gene89249	K00799 glutathione S-transferase [EC:2.5.1.18]|(GenBank) glutathione S-transferase, amino-terminal domain protein (A)
magenta	MS.gene94273	K00799 glutathione S-transferase [EC:2.5.1.18]|(GenBank) glutathione S-transferase, amino-terminal domain protein (A)
magenta	MS.gene98227	K00235 succinate dehydrogenase (ubiquinone) iron-sulfur subunit [EC:1.3.5.1]|(GenBank) succinate dehydrogenase [ubiquinone] iron-sulfur subunit (A)

## Data Availability

The original contributions presented in the study are included in the article and Supplementary Materials, further inquiries can be directed to the corresponding author.

## Supplementary Materials

The following supporting information can be downloaded at: https://www.mdpi.com/article/10.3390/foods13142261/s1, Figure S1: displays the soft threshold selection diagram and module-level clustering tree diagram; Table S1: Detailed Specifications of Biochemical Reagents Used in the Experiments; Table S2: Differentially Expressed Genes and Their Functional Classification.
