# Supplementary material for: Effects of Selenium Content on Growth, Antioxidant Activity, and Key Selenium-Enriched Gene Expression in Alfalfa Sprouts
Source: Foods. 2024 Jul 18;13(14):2261. doi: 10.3390/foods13142261 (PMC11276560; doi:10.3390/foods13142261)
Supplement: Supplementary file 1 [file foods-13-02261-s001.zip › Figure S1.pdf]

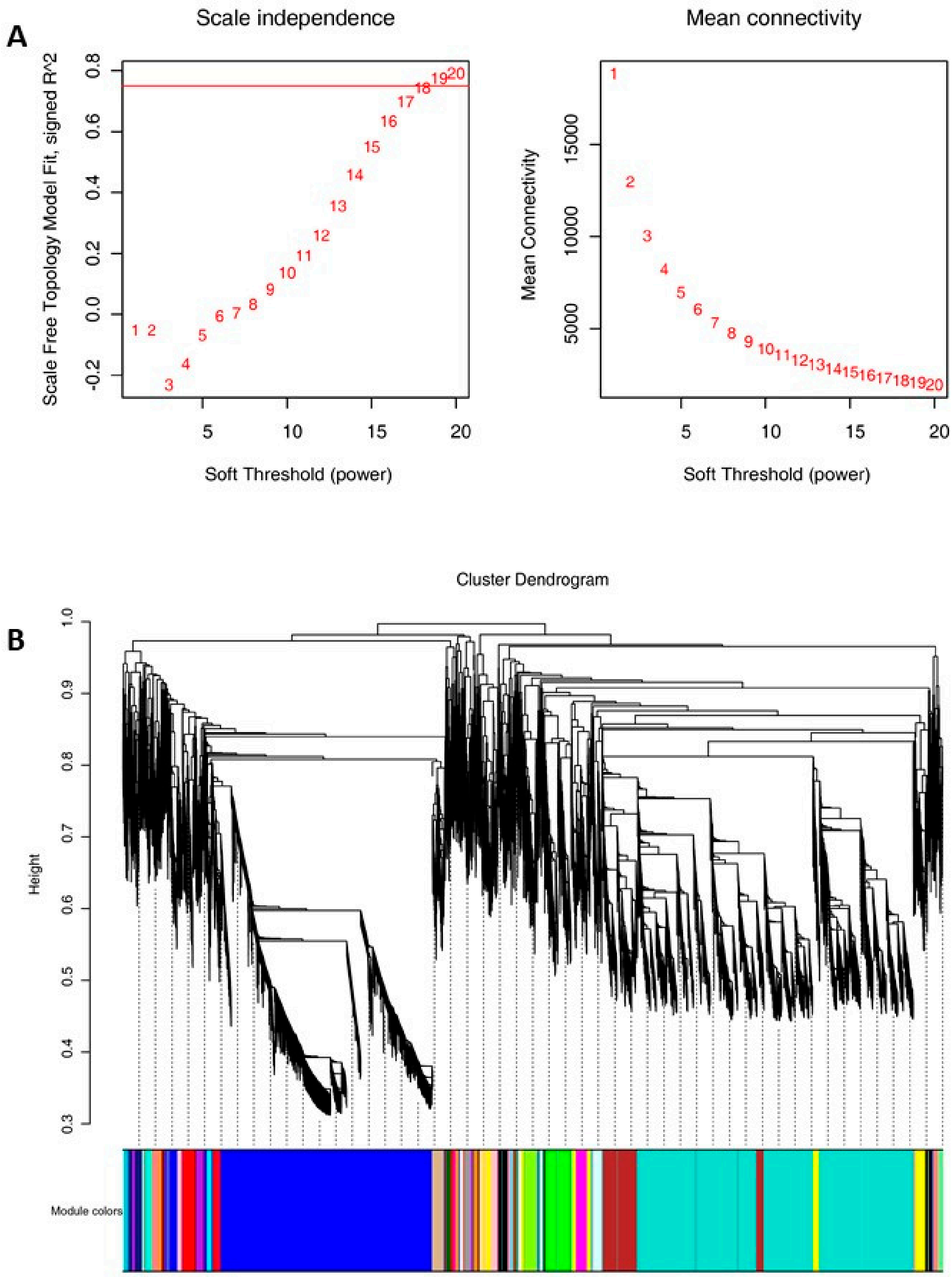

Figure S1. displays the soft threshold selection diagram and module-level clustering tree diagram. (A) The horizontal axis in the figure represents the weight parameter β, which acts as the soft threshold. The vertical axis on the left represents the square of the correlation coefficient in the corresponding network. A higher square of the correlation coefficient indicates that the network is closer to a scale-free distribution. We have set the threshold of the square of the correlation coefficient to be 0.85. The vertical axis on the right represents the mean of all gene adjacency functions in the corresponding gene module. The optimal beta value is the soft threshold used for subsequent analysis. (B) Each color in the figure corresponds to a gene in the clustering tree, indicating that they belong to the same module. If certain genes consistently exhibit similar expression changes in a physiological process or in different tissues, it suggests a functional relationship between them. These genes are

defined as a module in the upper part of the tree. The vertical distance in the diagram represents the distance between two nodes (genes), while the horizontal distance is not meaningful.
